# Design and Instrumentation of a Measurement and Calibration System for an Acoustic Telemetry System

**DOI:** 10.3390/s100403090

**Published:** 2010-03-31

**Authors:** Zhiqun Deng, Mark Weiland, Thomas Carlson, M. Brad Eppard

**Affiliations:** 1 Pacific Northwest National Laboratory, P.O. Box 999, Richland, WA 99332, USA; E-Mails: Mark.Weiland@pnl.gov (M.W.); thomas.carlson@pnl.gov (T.C.); 2 U.S. Army Corps of Engineers, Portland District, P.O. Box 2946, Portland, OR 97208, USA; E-Mail: Matthew.B.Eppard@usace.army.mil

**Keywords:** underwater transducers, piezoelectric sensors, acoustic telemetry

## Abstract

The Juvenile Salmon Acoustic Telemetry System (JSATS) is an active sensing technology developed by the U.S. Army Corps of Engineers, Portland District, for detecting and tracking small fish. It is used primarily for evaluating behavior and survival of juvenile salmonids migrating through the Federal Columbia River Power System to the Pacific Ocean. It provides critical data for salmon protection and development of more “fish-friendly” hydroelectric facilities. The objective of this study was to design and build a Measurement and Calibration System (MCS) for evaluating the JSATS components, because the JSATS requires comprehensive acceptance and performance testing in a controlled environment before it is deployed in the field. The MCS consists of a reference transducer, a water test tank lined with anechoic material, a motion control unit, a reference receiver, a signal conditioner and amplifier unit, a data acquisition board, MATLAB control and analysis interface, and a computer. The fully integrated MCS has been evaluated successfully at various simulated distances and using different encoded signals at frequencies within the bandwidth of the JSATS transmitter. The MCS provides accurate acoustic mapping capability in a controlled environment and automates the process that allows real-time measurements and evaluation of the piezoelectric transducers, sensors, or the acoustic fields. The MCS has been in use since 2009 for acceptance and performance testing of, and further improvements to, the JSATS.

## 1. Introduction

The Juvenile Salmon Acoustic Telemetry System (JSATS) is an active sensing technology that was developed by the U.S. Army Corps of Engineers (USACE), Portland District, to evaluate the migration timing, behavior, and survival of juvenile salmonids migrating through the Federal Columbia River Power System (FCRPS) [1]. The JSATS consists of acoustic microtransmitters (or transducers); autonomous, cabled, or portable receivers with hydrophones (piezoelectric sensors); and data management and processing applications. Receivers have been deployed at large hydroelectric dams [2] and in free-flowing sections [3] of the FCRPS, as well as throughout the estuary of the Columbia River [4]. Each microtransmitter, surgically implanted in study fish, transmits a unique binary code encoded using binary phase shift keying (BPSK) at a frequency of 416.7 kHz. Its code structure includes a total of 31 bits, with 7 synchronization bits (Barker code 1110010), 16 data bits, and 8 cyclic redundancy check (CRC) bits. The microtransmitters emit this unique 31-bit code at a programmed interval, typically every 3, 5, or 10 s, for 310 cycles at a carrier period of 2.4 μs, resulting in an acoustic length of approximately 1.1 m at 20 °C.

When the study fish implanted with the microtransmitters travel within the detection range of the deployed receivers, the acoustic signals are converted into an analog voltage by the piezoelectric sensors in the receivers [5,6], amplified by high-impedance preamplifiers, and transmitted to a signal-conditioning interface where they are further amplified and conditioned prior to being input to a digital signal processing (DSP) and field-programmable gate array (FPGA) card for detection analysis. The conditioned and detected signals are then decoded by BPSK, a digital modulation technique that transmits and decodes messages by altering the phase of the carrier wave using two phases, 0 and 180° [7]. Self-contained autonomous receivers that detect, decode, and store decoded messages are typically deployed in areas within the Columbia River basin where a power source is not readily available and cable runs are not feasible. Cabled systems are deployed at hydroelectric facilities and are used to determine route of passage and near-dam behavior for tagged fish. Each cabled system is synchronized to a universal Global Positioning System (GPS) clock with accuracy within 0.4 μs, and the acquired waveforms are saved to the computer before being decoded. Similar to GPS by satellites, time-of-arrival information from at least four receivers is required to resolve three-dimensional source locations [8,9].

The detailed information of fish movements and survival that the JSATS provides is critical to understanding the effects of hydroelectric systems on salmonid stocks listed under the Endangered Species Act [1]. However, before the JSATS is deployed in the field, it requires comprehensive acceptance and performance testing in a controlled environment. The objective of this study was to design and build a Measurement and Calibration System (MCS) for evaluating and further improving the JSATS.

## 2. System Instrumentation

**Figure 1.**
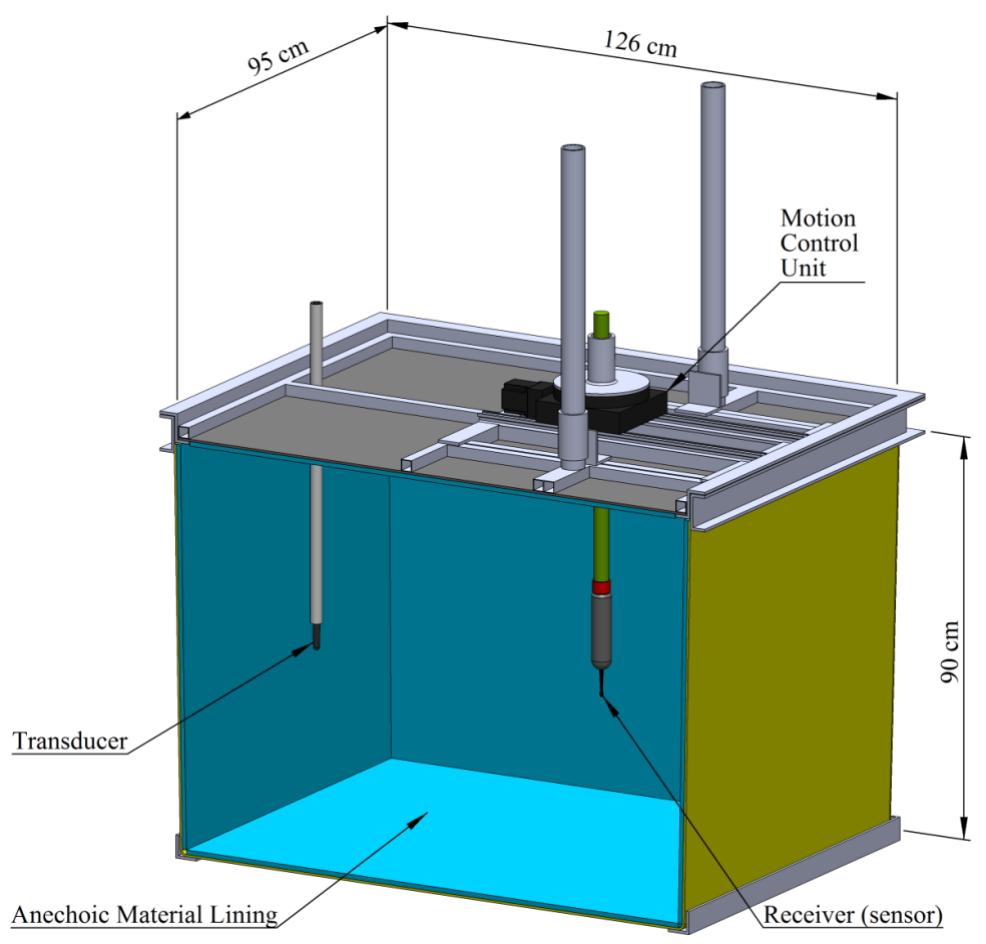
Schematic of the measurement tank for receiver (sensor) sensitivity and beam pattern testing.

**Figure 2.**
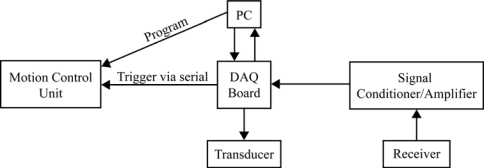
Functional diagram of the measurement and calibration system.

The MCS consists of reference transducers, a water test tank lined with anechoic material (Figure 1), a motion control unit, reference receivers, a signal conditioner and amplifier unit, data acquisition (DAQ) board, a MATLAB control interface, and a computer. Because the system employs the computer’s DAQ board to generate the waveforms, the waveform simulator/generator is not shown in the functional diagram (Figure 2).

The rectangular fiberglass water test tank is 1.26 m in length, 0.95 m in width, and 0.90 m in depth. It is lined with 26-mm-thick anechoic material (acoustic absorber Aptflex F48, Precision Acoustics, Ltd, Dorchester, Dorset, UK). This special material has low ultrasonic reflection and is highly absorbent for ultrasound. For a 26-mm-thick lining, the echo reduction is better than 20 dB (10% of reflected magnitude) for acoustic waves at frequencies below 500 kHz, resulting in accurate underwater acoustic measurements without impact from acoustic noise in a controlled environment.

A broad band spherical hydrophone (RESON TC4034, RESON A/S, Slangerup, Denmark) or universal high-frequency transducer (RESON TC3029) is used as the reference transducer to transmit the encoded acoustic signals by converting the voltage signal to acoustic pressure. The TC4034 provides uniform omnidirectional characteristics over a wide usable frequency range of 1 Hz to 480 kHz. The TC3029 is a compact universal 500 kHz transducer, ideal for high-frequency measurements. The transducer is connected to an analog output channel of the DAQ board.

A miniature probe hydrophone (RESON TC4038) is used as the reference receiver. This hydrophone provides a flat frequency response and omnidirectional characteristics for high frequencies ranging from 100 to 500 kHz. Analog signals from the receivers are conditioned by a low-noise 1 MHz bandwidth voltage preamplifier (RESON EC6081/VP2000). The low-pass and high-pass filters (−6 dB/octave roll-off) are set typically at 1 MHz and 50 kHz, respectively. The amplifier gain ranges from 0 to 60 dB to optimize the input range of the DAQ board, depending on the amplitude of the incoming signal.

The interface was developed in MATLAB computing language (MathWorks, Inc., Natick, Massachusetts, USA) for a Dell Dimension 340 desktop computer (Dell Inc., Round Rock, Texas, USA) to communicate with all components, including the motion control unit, waveform simulation/generation, data acquisition, and data analysis. The MATLAB control and analysis interface could also be compiled into libraries and executable files for use in the field or by any users without access to MATLAB. A NI PCI-6111 board (National Instruments Corporation, Austin, Texas, USA) was used for both outputting the encoded signals and acquiring the received signals. It is a 12-bit analog-to-digital, simultaneous-sampling multifunction data acquisition board. It has two simultaneously sampled analog inputs at 5 million samples per second (MS/s) per channel and two 16-bit analog output channels at up to 4 MS/s for one channel or 2.5 MS/s for dual channels. It was chosen for its high output and sampling rate capabilities and compatibility with MATLAB. The high sampling rates allow accurate representation of JSATS 416.7 kHz signals with at least ten samples per signal cycle. The compatibility with MATLAB allows easy incorporation of data analysis packages already developed in MATLAB, leading to real-time measurement and data analysis.

The motion control unit consists of various automation components (Parker Hannifin Corp., Cleveland, Ohio, USA): a 20802 rotary table geared with an HV231 stepper motor, as well as an E-AC motor drive and a 6K8 controller. The receiver is held in the tank by a cylindrical fixture extending to the motorized rotary positioning table above, which is attached to the top of the tank (Figure 1). The controller communicates with the computer *via* a serial cable and MATLAB commands.

## 3. System Calibration and Data Acquisition

During the design process, a general formulation was derived for system calibration and performance measurements. Suppose:

- V_rms_ is the root-mean-square (RMS) value of the transmitted waveform in volts.
- W_rms_ is the RMS value of the acquired waveform in volts.
- G is the corresponding gain of the signal amplifier in decibels (dB), defined as 20 times the logarithm of the RMS ratio.
- D is the distance from the transducer to the receiver in meters; D is 1 m in Figure 1.
- Ps is the transmitting sensitivity of the transducer in dB re 1 μPa /V @ 1 m.
- Hs is the hydrophone sensitivity in dB re 1 V /μPa.
- TL is the transmitting loss in dB.

Transmitting loss is estimated [10] as

(1) TL≈20×log(D)

If the transducer sensitivity is known, the receiver (hydrophone) sensitivity is then obtained from

(2) $$\text{Hs}=20\times \text{log}\left(W_{\text{rms}}\right)-20\times \text{log}\left(V_{\text{rms}}\right)-\text{Ps}+\text{TL}-G$$

If the receiver sensitivity is known, then transmitting sensitivity of the transducer is

(3) $$\text{Ps}=20\times \text{log}\left(W_{\text{rms}}\right)-20\times \text{log}\left(V_{\text{rms}}\right)-\text{Hs}+\text{TL}-G$$

The system was designed to automate the process that provides real-time measurements and evaluation of the piezoelectric transducers, sensors, or acoustic fields. A basic functional breakdown is as follows:

1. The DAQ board triggers the waveform generator to send a preprogrammed signal (pulse, sine wave, or full coded waveform) to the transducer.
2. The transducer converts the signal to acoustic pressure signals in the tank.
3. The piezoelectric sensor in the receiver converts the acoustic pressure signal into voltage that is conditioned and amplified before being digitized and loaded into the computer buffer memory.
4. The signal is saved to the computer hard drive if it meets the predetermined detection criteria.
5. Steps 1 through 4 are repeated until the preset number of measurements is made.
6. The data acquisition board triggers the motion control unit to move to the next location and start new measurements.
7. Decoding utilities are used to evaluate system performance; data analysis packages are employed to compute sensitivities, beam profiles, or other characteristics.

**Figure 3.**
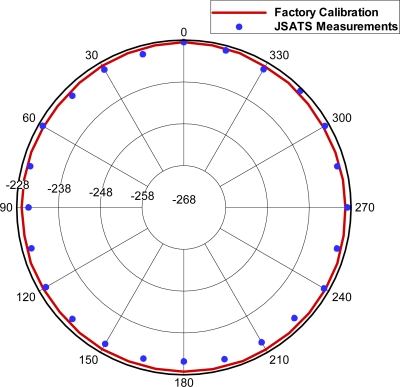
Comparison of the beam profile measured using the measurement and calibration system and factory-calibrated data.

To calibrate the system, the reference transducer and reference receiver were spaced 1.12 m apart in the tank. The output and data acquisition sampling frequency was set to 2.5 MS/s. As an example, the reference hydrophone (RESON TC4038) had a factory-calibrated sensitivity of −228.50 dB re 1V /μPa at a carrier wave frequency of 416.7 kHz. Ps was 161.3 dB re 1 μPa /V @ 1 m. For this test case, the transmitted waveform had an RMS value of 5 V. Gain was set as 50 dB. Eight measurements were taken 1-s apart for each orientation, with RMS values between 0.617 and 0.618 V (SE is 0.0004 V) at the position with the highest sensitivity, which was defined as the zero-degree orientation. The measured hydrophone sensitivities at zero degree using Equation (2) ranged from −228.49 to −228.47 dB re 1 V/μPa (SE is 0.01), which agrees well with the factory-calibrated data (−228.50 dB re 1 V/μPa). The beam profile also demonstrated good agreement (Figure 3).

## 4. System Performance Measurements

**Figure 4.**
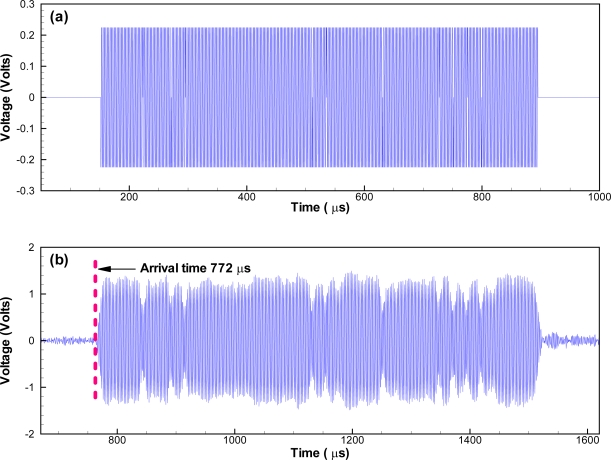
Comparison of original simulated waveform and acquired waveform. Note that there is a slight delay due to the travel time of the signal and latency of hardware triggering. However, this delay has no impact on the measurements because only the message portion of the waveform is used for analysis.

The test tank was designed to provide accurate acoustic mapping capability in a controlled environment. It was verified by transmitting a coded waveform (720087AF) at a signal level of 126 dB re 1 μPa @ 1 m using the transducer (Figure 4a). It was equivalent to 30 dB attenuation, given the typical source level of 156 dB re 1 μPa @ 1 m for JSATS microtransmitters. An analogous range between the transducer and the receiver would be 32 m given 30 dB = 20 × log(32). The waveform acquired by the receiver had a signal-to-noise ratio of 24.2 dB and showed no evidence of impact from acoustic reflection (Figure 4b). It was decoded successfully with correct time of arrival at 772 μs.

**Figure 5.**
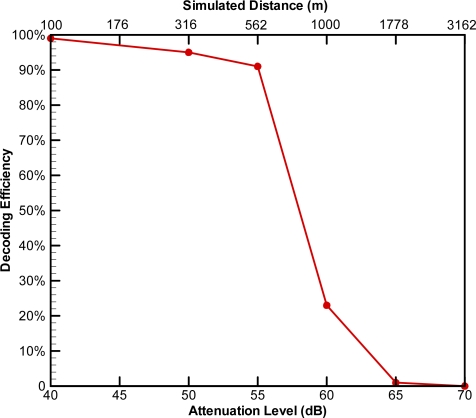
Decoding efficiency for simulated range testing in an ideal environment.

Because JSATS transmitter codes use 16 data bits for four hexadecimal characters and CRCs are dependent on the four data characters, there are 65,536 unique tag code combinations for JSATS code space. Phase shift count was defined as the number of phase changes in the binary representation of the 16 data bits and 8 CRC bits. For example, for tag code 72007058 the data are hexadecimal 0070 and the CRC is hexadecimal 58, so the data bits and CRC bits (excluding 72—the 7 bit barker code) are 0000-0000-0111-0000-0101-1000, and the phase shift count is 6. Twenty-eight tag codes were used to represent the 14 different phase shift counts of JSATS code space, with two tag codes randomly selected from each phase shift count category. Decoding efficiency was calculated as the number of correctly decoded detections divided by the number of actual transmissions. An attenuation curve was created by averaging the decoding efficiencies of the 28 tag codes for each of the six levels of signal attenuation (−40, −50, −55, −60, −65, −70 dB). These attenuation levels were achieved by transmitting the encoded waveforms at signal levels of 116, 106, 101, 96, 91, and 86 dB re 1 μPa @ 1 m, respectively. For simulated ranges in an ideal environment, they are equivalent to 100, 316, 562, 1,000, 1,778, and 3,162 m, respectively. For each attenuation level, 560 encoded signals were transmitted. The measurement results confirmed that the receiver had a detection range of more than 500 m in a controlled freshwater environment (Figure 5). The current JSATS receiver design requires that a receiver demonstrates a decode efficiency at the −55 dB level greater than or equal to 50% before it is accepted and deployed for field use.

**Figure 6.**
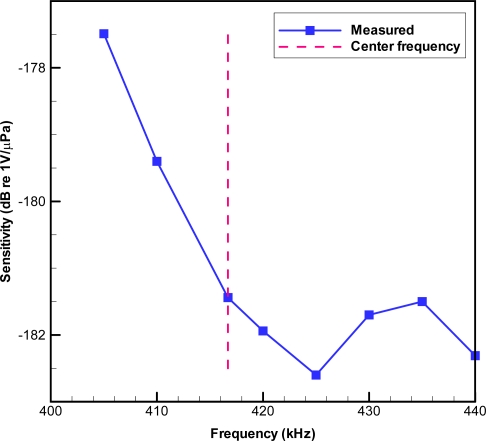
Sensitivity measurements of receivers in the measurement tank at different frequencies.

Encoded signals were transmitted *via* the reference transducer to the receiver using carrier wave frequencies different from the JSATS center frequency. The frequency shift was achieved by re-sampling the original waveform using a polyphase filter implementation [11]. Figure 6 shows a typical example of the sensitivity measurement within a frequency bandwidth. For this example, the measured sensitivities were within specified −180 ± 3 dB re 1 V/μPa across the full range of the frequency bandwidth for the JSATS transmitter (416.7 kHz ± 0.5%).

## 5. Conclusions

The JSATS is an active sensing technology that was developed by the USACE for evaluating behavior and survival of juvenile salmonids migrating through the FCRPS. It has been successfully used to acquire information for salmon protection and development of more “fish-friendly” hydroelectric facilities. The MCS was designed and implemented to provide accurate acoustic mapping capability in a controlled environment, and automates the process that provides real-time measurements and evaluation of the JSATS piezoelectric transducers or sensors or the acoustic fields. The fully integrated MCS has been evaluated successfully using different encoded signals at various simulated distances and frequencies within the bandwidth of the JSATS transmitters. The MCS is also currently being used for competitive procurement acceptance and performance testing and for further development and refinement of the JSATS.
